# Cropland Footprints of Australian Dietary Choices

**DOI:** 10.3390/nu12051212

**Published:** 2020-04-25

**Authors:** Bradley Ridoutt, Kim Anastasiou, Danielle Baird, Javier Navarro Garcia, Gilly Hendrie

**Affiliations:** 1 Commonwealth Scientific and Industrial Research Organisation (CSIRO) Agriculture and Food, Clayton South, Victoria 3168, Australia; 2 Department of Agricultural Economics, University of the Free State, Bloemfontein 9300, South Africa; 3 CSIRO Health and Biosecurity, Adelaide, South Australia 5000, Australia; kim.anastasiou@csiro.au (K.A.); danielle.baird@csiro.au (D.B.); gilly.hendrie@csiro.au (G.H.); 4 CSIRO Agriculture and Food, St. Lucia, Brisbane, Queensland 4067, Australia; javi.navarro@csiro.au

**Keywords:** biodiversity, dietary guidelines, diet quality, discretionary food, environmental impact, land use, life cycle assessment, sustainable diet

## Abstract

Food systems vitally depend on croplands, which are a scarce natural resource. Croplands are also heterogeneous, differing in productive capability and in environmental context. Some are in regions of high biodiversity conservation importance, others in regions vulnerable to food insecurity. In this study, life cycle assessment was used to quantify cropland scarcity footprints, cropland biodiversity footprints and cropland malnutrition footprints for 9341 individual Australian adult daily diets. Dietary cropland scarcity footprints averaged 7.1 m^2^yr-e person^−1^ day^−1^, exceeding a target of 6.1 m^2^yr-e person^−1^ day^−1^, consistent with the proposed global cropland planetary boundary of 15% of the ice-free land area. Discretionary foods, which are energy-dense and nutrient-poor foods high in saturated fat, added sugars and salt, and alcohol and are not essential to a healthy diet, made the largest contribution, followed by fresh meats and alternatives, breads and cereals, fruit, dairy and alternatives and vegetables. Around 45% of the variation in cropland footprint between individuals was explained by differences in total dietary energy intake. Diets characterised by higher diet quality and lower cropland scarcity footprint required only 4.2 m^2^yr-e person^−1^ day^−1^ and recommended diets based on the food choices of this subgroup required 5.9 m^2^yr-e person^−1^ day^−1^. Eating within the global cropland planetary boundary appears realistic if Australians greatly reduce their intake of discretionary foods and moderate their food choices within the “meat and alternatives” food group.

## 1. Introduction

Food-based dietary guidelines have now been developed in more than 100 countries [1]. Traditionally, these guidelines have been designed to support food consumption that is healthy and nutritionally adequate. However, there are now calls for dietary guidelines to evolve by taking on the additional objective of supporting patterns of food consumption that have lower environmental impact [2]. The food system is a major source of environmental impact [3], and there is scope for dietary change to complement other environmental improvement strategies such as adopting more efficient farming practices, reducing production losses and avoiding food waste [4]. Consequently, the number of studies addressing the subject of sustainable diets has grown rapidly [5,6,7,8], although it has been noted that the evidence base is skewed toward the assessment of dietary greenhouse gas emissions [9], with less evidence in relation to other environmental aspects [10].

The food system is critically dependent upon croplands. However, croplands are a scarce natural resource and the conversion of forests and grasslands into new cropland is linked to losses of biodiversity and other ecosystem services [11,12,13,14]. Recent estimates suggest that croplands occupy almost 1.9 billion hectares globally [15], which is around 14% of the total ice-free land area. Furthermore, the area of cropland is projected to continue to expand [16,17], especially in biodiverse tropical regions [18,19,20], to meet the demands for food, bioenergy and other bio-products of an increasing world population [21]. To guard against major and potentially irreversible earth system change, nine planetary boundaries have been proposed, including a boundary for the global extent of croplands at 15% of the ice-free land surface [22,23]. This boundary may be transgressed soon, if not already, highlighting the urgency of strategic action to limit further cropland expansion.

The issue, however, is that croplands are not homogeneous. Croplands can vary greatly in productive capability due to differences in climate, topography and soil characteristics [24]. They can also have greatly differing impacts on biodiversity, with some cropland located in areas of high conservation importance due to high species richness or the occurrence of endangered or critically endangered species [11,18,19,25]. In addition, some croplands are located in regions where food security is poor and local communities are vulnerable to malnutrition [26]. What this means is that a simple area-based assessment of cropland used to produce a product or to sustain a dietary pattern is not a reliable indicator of environmental impact [27]. For example, cereals grown on cropland of lower productivity may have a higher cropland utilization per kg of output than cereals grown on cropland of higher inherent productivity; but this does not mean they are less sustainable or contribute in a greater way to cropland scarcity or biodiversity decline [28,29,30,31]. Instead, environmental indicators are necessary that address the specific environmental concerns related to cropland use [32,33], consistent with the international standards that govern life cycle assessment (LCA) [34,35].

In this study, we use LCA to model cropland footprints related to productive land scarcity, malnutrition-related health impacts and biodiversity loss for 9341 individual Australian adult daily diets obtained from the Australian Health Survey [36]. Our objective was to identify dietary patterns existing within the Australian community that are characterised by higher diet quality and lower environmental impacts related to cropland use. Land use and biodiversity indicators have previously been applied to average diets and dietary scenarios [4,37,38,39,40,41,42,43,44,45,46,47]. To our knowledge, this is the first study to report on the impacts related to cropland use associated with individual daily diets. Our study design is based on the knowledge that there is enormous diversity in dietary habits existing within the Australian community already [48] and that dietary shifts of any significant proportion are unlikely to occur beyond the existing range. Our purpose is to expand the evidence base concerning healthy sustainable diets and support potential future amendment of food-based dietary guidelines.

## 2. Materials and Methods

### 2.1. Dietary Intake Data

The Australian Health Survey [36] was a large, nationally representative health survey undertaken by the Australian Bureau of Statistics (ABS) that involved around 50,000 adults and children. For this investigation into cropland footprints, dietary intake data were obtained from the National Nutrition and Physical Activity Survey component [48] which included 9341 adults. The survey employed a complex sampling method [36] to enable the estimation of dietary intake for the Australian population as well as demographic subgroups through application of population weighting factors. Dietary intake data were collected using a 24-h recall process and involved trained interviewers. The data included all foods and beverages and portion sizes consumed on the day prior to the interview. Data were collected across all days of the week and over a 13-month period to account for seasonal variation in eating habits. The dietary intake data included 5645 individual foods, many finely delineated, to enable comprehensive analysis of nutrient intakes. There was an under-representation of dietary intakes recalled for Friday and Saturday (due to interviews needing to be conducted on Saturday and Sunday), which was accounted for with an additional weighting factor.

To enable integration of the dietary intake data and cropland footprint data, processed foods and mixed dishes were disaggregated into basic components as described previously [49], applying archetypal recipe files published by the ABS [36]. Conversion factors, obtained predominantly from a reference database managed by Food Standards Australia and New Zealand [50], were used to translate cooked food portions into raw quantities.

Under-reporting of food intake is a consideration of all dietary intake surveys. This can be the result of inaccurate recall or intentional misreporting of foods and portion sizes eaten. To support interpretation of the nutrition survey data, the ABS has estimated the prevalence of under-reporting [36]. These factors of 21% for females and 17% for males were uniformly applied to the dietary intake data. It is possible that under-reporting of food intake was biased towards certain types of energy-dense foods and individuals of particular weight status [51,52]. However, a reliable method to differentially adjust for under-reporting is not presently available. Adjustment for under-reporting was necessary to avoid systematic underestimation of dietary cropland footprints and to enable comparison between reported and recommended diets.

For each of the 9341 adults included in the nutrition survey, dietary intake was expressed in terms of energy intake and number of servings of each of the food groups described in the Australian Dietary Guidelines [53]. Beverages that are not included within the official food groups, such as tea and coffee, formed a separate category. Different meats and alternatives were separately examined due to the focus on land use associated with meat production appearing in the literature [10,54,55,56]. Beef and lamb were grouped as they share similar production systems in Australia, have similar cropland scarcity footprints and are both considered red meat within the Australian Dietary Guidelines [53]. Also, different types of discretionary foods were itemised due to the prevailing public health nutrition concerns about their excessive consumption. Discretionary foods are energy-dense and nutrient-poor foods high in saturated fat, added sugars and salt, and alcohol, which contribute around a third of dietary energy intake. Using the ABS population weighting factors, the mean dietary intake for Australian adults was calculated, as well as mean values for age and gender subgroups defined in the Australian Dietary Guidelines (i.e., 19 to 50 years, 51 to 70 years and 71 years and above).

### 2.2. Dietary Quality Analysis

For each of the 9341 adult daily diets, a diet quality score was determined by application of the food-based Dietary Guideline Index (DGI) [57], as described previously [49]. The DGI provides a score out of 100 that reflects overall compliance with the Australian Dietary Guidelines [53]. A higher score reflects greater compliance.

### 2.3. Cropland Footprint Modelling

Cropland footprint modelling was undertaken using LCA. Initially, cropland footprint data for Australian agricultural commodities were obtained from a previous study [31]. This included Australian-produced livestock products, taking into account the cropland footprints associated with feed rations. Australia is a net exporter of most agricultural commodities and it has been estimated that greater than 90% of available food is Australian-grown [58,59]. This data source [31] also describes the cropland footprints of the main agricultural commodities that are imported because they are not grown in Australia to any significant extent, namely coffee bean, cocoa bean, tea, coconut, hazelnut, hops and oil palm fruit. In brief, these data were developed using a 1.1 km^2^ spatial resolution map of agricultural production including crop yield information and involved the application of three life cycle impact assessment models, described below. Detail is available in the associated reference [31]. The cropland footprints of individual foods were quantified using conversion factors that translate agricultural commodities into retail products and edible portions, as described previously [49]. Former cropland areas occupied by food-processing factories, transportation systems and other infrastructure were deemed not materially important and were excluded from the assessment.

Three cropland footprint indicators were quantified for each individual adult daily diet, reflecting different environmental concerns relating to cropland occupation. Firstly, a cropland scarcity footprint (CSF) was quantified. Cropland is a globally finite and scarce natural resource and the occupation of cropland contributes to this scarcity as cropland used for one productive purpose cannot be used for another. However, not all cropland is equally productive. Therefore, CSFs were quantified taking into account productive capability using the net primary productivity of potential biomass at each location, reflecting the natural capability of the land. CSF results were expressed in m^2^ yr-e (equivalent), with cropland of global average productivity as the reference. Secondly, a cropland biodiversity footprint (CBF) was quantified using the biodiversity impact factors of Chaudhary and Brookes [60]. These impact factors report potential species loss based on 5 taxa in 804 ecoregions of the world. CBF results were expressed as potentially disappeared fraction of species (PDF). Thirdly, a cropland malnutrition footprint (CMF) was quantified using the impact factors of Ridoutt et al. [26]. These impact factors report potential protein-energy malnutrition impacts considering potential domestic and trade-related food deficits arising from cropland occupation. The factors are highest in countries where protein-energy malnutrition is prevalent and in countries that share a trade relationship with these regions. As is typical in LCA, the factors express the potential impact of production (i.e., occupying the cropland) and not the potential benefits of use (i.e., food consumption). Crop products have the potential to be used in numerous ways: for direct human consumption, for livestock rations, for biofuels or other industrial products. Even when crop products are intended for human consumption, they may be wasted or contribute to energy intakes that exceed a healthy diet. CMF results were expressed in disability-adjusted life years (DALYs). To calculate cropland footprint results, each spatially explicit instance of cropland occupation was multiplied by the spatially relevant impact factor. Cropland footprint results for almost 150 separate food items are presented in the Supplementary Materials, Table S3.

### 2.4. Dietary Pattern Modelling

For the 5157 adult daily diets in the 19–50-year age grouping, quadrant analyses were performed by ranking individual diets by diet quality score and each of the cropland footprints. Separate quadrant analyses were performed for each type of cropland footprint. After excluding daily diets within 0.25 standard deviations of the mean for each parameter, diets with higher diet quality scores and lower cropland footprints (better diets; HDQ-LCF) were compared to diets with lower diet quality scores and higher cropland footprints (poorer diets; LDQ-HCF). Comparisons were also made between average adult diets and diets based on the recommended number of servings of each food group as described in the Australian Dietary Guidelines [53]. Here, it is important to note that the Australian Dietary Guidelines are not prescriptive in terms of specific food choices within a food group. For example, women in the 19–50-year age group are recommended to consume five servings of vegetables per day. Apart from highlighting the benefits of choosing a variety of colours of vegetables, there is no recommendation about which specific vegetables should be eaten. As such, it is possible to construct a range of specific diets which are equally compliant with the Australian Dietary Guidelines. For the purpose of this study, cropland footprint results for the recommended diet were based on the cropland footprint intensity of each food group in the average diet. As a further analysis, the cropland footprint intensity of each food group in the “better diets” (described above) was also used. Potential cropland footprint reductions from food choice within a food group were individually considered.

### 2.5. Other Descriptive Statistics

The Pearson correlation coefficient was used to explore the degree of relationship between the three sets of cropland footprint results and dietary energy intake (*n* = 9341). Using data from Hendrie et al. [61] for dietary greenhouse gas emissions and Ridoutt et al. [49] for dietary water scarcity footprint, the Pearson correlation coefficient was also used to explore the relationship between dietary cropland footprints and these footprints (*n* = 9341). Correlations between footprints were undertaken after controlling for variation in dietary energy content. This is because dietary environmental footprint results are correlated with total dietary energy intake.

## 3. Results

### 3.1. Cropland Footprint and Energy Intake

**Figure 1:**
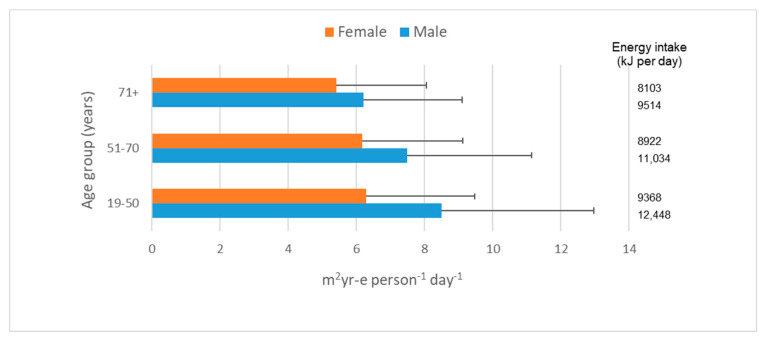
This cropland scarcity footprint of Australian adult diets based on 9341 individual daily diets reported in the Australian Health Survey. Error bars indicate the standard deviation.

The cropland scarcity footprints of Australian adult daily diets averaged 7.09 m^2^yr-e per person. They were generally lower for females (6.15 m^2^yr-e per person) than males (8.02 m^2^yr-e per person; Supplementary Table S4) and generally lower for older adults such as those in the 71 years and above age category (Figure 1). In part, this is explained by differences in overall energy intake. Males in the 19–50-year age group had the highest average cropland scarcity footprint (8.49 m^2^yr-e per person) as well as the highest average energy intake (12,448 kJ per day). Females in the 71 years and above age category had both the lowest average cropland scarcity footprint (5.40 m^2^yr-e per person) and lowest average energy intake (8103 kJ per day). Overall, variation between individuals in dietary energy intake explained around 45% of the variation in dietary cropland scarcity footprint (R^2^: Females 0.43, Males 0.47). This is a marginally stronger linear relationship than has previously been reported in Australia for dietary greenhouse gas emissions and for dietary water scarcity footprints whereby around one-third of the variation was explained by variation in dietary energy intake [49,61]. In the case of cropland biodiversity footprint and cropland malnutrition footprint, the average values for Australian adult daily diets were 1.19 × 10^−12^ PDF per person and 2.82 × 10^−5^ DALY per person, respectively (Supplementary Table S4). These cropland footprint results also varied by gender and age group, reflecting again, in part, variation in total energy intake (cropland malnutrition footprint R^2^: Females 0.40, Males 0.43; cropland biodiversity footprint R^2^: Females 0.34, Males 0.32).

It is not easy to find comparable results in the literature. Osei-Owusu et al. [62] reported cropland requirements for food supply in Denmark of 1282 kha in 2013, which equates to around 6.3 m^2^ per person per day. This value is lower than the average cropland scarcity footprint of Australian adults reported in this study. However, the Danish value includes children, many of whom would have lower dietary energy intake than adults. Also, the Danish study did not consider the productivity of the cropland that was occupied. If cropland with productivity above the global average was used, this would also contribute to a lower value. An even lower cropland requirement was reported in the UK (4.9 m^2^ per person per day; [63]). However, for the same reasons just mentioned, it is not possible to make any direct comparison to cropland scarcity footprint. In a study of dietary patterns in 16 European countries, de Ruiter et al. [37] reported cropland requirements varying from 5.7 to 12.2 m^2^ per person per day and noted that cropland requirements were higher in southern Europe where agricultural lands are drier and yields tend to be lower, reinforcing the point made above. Regarding biodiversity impacts due to food consumption, Crenna et al. [46] evaluated a basket of representative food products in Europe. However, a different biodiversity impact assessment model was used, meaning that the values reported are not directly comparable with this study. Chaudhary et al. [40] evaluated the food-related per capita biodiversity footprints for 156 counties. However, results are presented as a normalised score (0–100), again meaning that no direct comparison is possible with the Australian values. Regarding cropland malnutrition footprint, to our knowledge, our results are completely novel.

### 3.2. Contribution Analysis

Cropland footprint results were disaggregated according to the food groupings described in the Australian Dietary Guidelines [53]. The highest contribution came from discretionary foods (Table 1). These foods, sometimes alternatively referred to as non-core foods or indulgence foods [64], are energy-dense and nutrient-poor foods high in saturated fat, added sugars and/or salt, and alcohol [53]. There are very many varieties of these discretionary foods available within the Australian food system and addressing the excessive consumption of these foods is a major focus for public health nutrition professionals [65] as they can displace intake of core foods and contribute to weight gain and the development of chronic health conditions [66]. Across the three cropland footprints, discretionary foods contributed between 33% and 36% (Table 1).

The second largest contribution to cropland footprints was from fresh meat and alternatives, 23.9% to 27.4% (Table 1). This food group in the Australian Dietary Guidelines [53] includes meats that have not undergone any preserving process, as well as eggs, tofu, legumes and other vegetarian and vegan alternatives to meat. Within this food group, the highest contribution came from poultry (9.5% to 11.7%), which is a popular variety of meat consumed in Australia [67], followed by beef and lamb, vegetarian alternatives, pork and seafood (Table 1). Breads and cereals made the next highest contribution, at around 12% of the total cropland scarcity footprint, followed by fruit and then dairy products and alternatives (Table 1). Although tea and coffee have some of the highest cropland footprints per kg of crop product [31], beverages made only a modest contribution to the total dietary cropland footprint (1.5% to 3.8%). That said, it is to be noted that according to the Australian Dietary Guidelines [53], some beverages are included in other food groups. For example, naturally and or artificially sweetened beverages, including soft drinks (or soda), fruit juice drinks and alcoholic beverages are considered discretionary foods. Milk and cereal beverages are included within the dairy and alternatives food group. Freshly squeezed fruit juice and beverages prepared from fruit juice concentrate without added sweetener are considered servings of fruit. Further detail concerning the breakdown of dietary cropland footprints by age and gender subgroups is provided in Supplementary Tables S5–S7.

### 3.3. Cropland Footprint and Diet Quality

**Figure 2:**
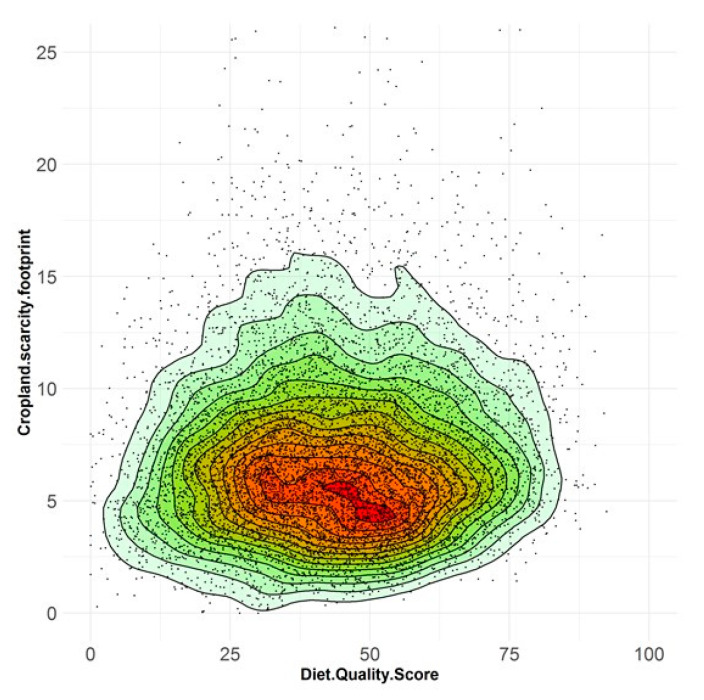
Cropland scarcity footprint (m^2^ye person^−1^ day^−1^) and diet quality score (range 0–100). Scatterplot showing diversity of individual adult daily diets reported in the Australian Health Survey (*n* = 9341). Shading indicates density of points from high (red) to low (green).

Individual daily diets were found to vary widely in both cropland footprint and diet quality score (Figure 2 and Supplementary Figure S1), with very little relationship between the two dimensions (Pearson correlations of 0.05 or lower). For the largest adult subgroup (19 to 50 years, *n* = 5157), the higher diet quality/lower cropland scarcity footprint subgroup (HDQ-LCF; *n* = 824) was compared to the lower diet quality/higher cropland scarcity footprint subgroup (LDQ-HCF; *n* = 832). The differences between these subgroups were very large, with the HDQ-LCF subgroup having a 62% lower cropland scarcity footprint (4.21 m^2^yr-e per person per day compared to 11.17 m^2^yr-e per person per day; Table 2) and a diet quality score that was more than double (58.4 compared to 26.4, out of a possible 100). In terms of dietary pattern, the distinguishing difference between these two subgroups was the intake of discretionary food: 2.09 servings per day for the HDQ-LCF subgroup compared to 14.54 servings per day for the LDQ-HCF subgroup. Indeed, the cropland scarcity footprint from discretionary food consumption by the LDQ-HCF subgroup (5.61 m^2^yr-e per person per day; Table 2) exceeded the cropland footprint from all food intake by the HDQ-LCF subgroup.

For these two subgroups, the differences in intake of core foods were much smaller, yet still important. The HDQ-LCF diets included higher intake of fruit (0.62 servings per day), vegetables (1.90 servings per day), cereals (1.5 servings per day), seafood (0.28 servings per day) and vegetarian alternatives to meat (0.33 servings per day) (Table 2). Overall, the HDQ-LCF subgroup included fewer servings of fresh meat and alternatives (0.70 servings per day), mainly explained by a lower intake of poultry (0.93 servings per day) and pork (0.26 servings per day) (Table 2). Similar results were obtained for these two subgroups for cropland biodiversity footprint and cropland malnutrition footprint (Supplementary Tables S8 and S9).

### 3.4. Recommended Dietary Scenario

The current average diet of 19–50-year-old adults was also compared to a diet consistent with the Australian Dietary Guidelines [53]. The data show what is well known in Australia, that on average, adults should increase their intake of all the five core food groups, and greatly limit intake of discretionary foods (Table 3). In particular, the intake of vegetables should more than double, from the current 2.47 servings per day to 5.5 servings per day (5 servings in the case of 19–50-year-old women and 6 servings in the case of men). In addition, intake of dairy products and alternatives should increase by around 70%, and fruit by almost 45%. If the current average diet was scaled to conform to the guidelines, with increased core food intake and reduced discretionary food intake, the cropland scarcity footprint would increase marginally from 7.41 to 7.71 m^2^yr-e per person per day, an increase of around 4% (Table 3). The same transition would also lead to small increases in the cropland biodiversity footprint (around 1%; Supplementary Table S10) and cropland malnutrition footprint (around 2%; Supplementary Table S11). However, if a transition to the recommended diet occurred in line with the food choices evident in the HDQ-LCF dietary pattern (Section 3.3), the cropland scarcity footprint would reduce by around 20% to 5.96 m^2^yr-e per person per day (Table 3). As such, food choice within a food group has an important bearing on dietary cropland footprints, especially choice within the meat and alternatives food group. Recommended diets based on the HDQ-LCF dietary pattern had markedly higher intake of seafood, red meat and vegetarian alternatives, and markedly lower intake of poultry and pork (Table 3). Following this approach, transitions to the recommended diet also have the potential to reduce the cropland biodiversity footprint by 21% (Supplementary Table S10) and the cropland malnutrition footprint by 22% (Supplementary Table S11).

## 4. Discussion

### 4.1. Eating within the Cropland Planetary Boundary

If global croplands are to occupy no more than 15% of the ice-free land surface, their total area cannot extend beyond about 2 billion ha. For a future global population of 9 billion inhabitants, this means that, on average, per capita cropland requirements should not exceed 2226 m^2^ per year or around 6.1 m^2^ per day. That said, global croplands vary in inherent productive capability and areas of high-productivity cropland should not be compared directly to areas of low-productivity cropland. Consequently, the per capita availability of cropland should be expressed as 6.1 m^2^ per day relative to cropland of global average productivity. For this reason, in this study, cropland scarcity footprints were quantified for Australian adult diets considering both area and productive capability, with results expressed in the units m^2^yr-e. It was found that at 7.1 m^2^yr-e, Australian adult daily diets currently exceed the target. However, there is great diversity in dietary habits, and those diets characterised by higher diet quality score and lower cropland scarcity footprint required only 4.2 m^2^yr-e per day (Table 2). As such, many Australians are already eating in a manner consistent with the global cropland planetary boundary. Compared to poorer quality diets with higher cropland scarcity footprint, the major distinguishing feature was a much lower intake of discretionary foods.

Adult daily diets consistent with current Australian Dietary Guidelines [53] were also found to have a cropland scarcity footprint within the global cropland planetary boundary, but only if these diets were based on food choices evident in the higher diet quality/lower cropland scarcity footprint dietary pattern (5.96 m^2^yr-e per day; Table 3). In this regard, food choice within the “fresh meat and alternatives” food group was found to be important. Lower cropland scarcity footprint diets, compared to current average diets, contained a larger number of servings of seafood, red meat (beef and lamb) and vegetarian alternatives (eggs, legumes, tofu, etc.), and fewer servings of poultry and pork (Table 3). It is noted that this level of red meat intake is above the current level of consumption, but within the threshold described in the Australian Dietary Guidelines [53].

Eating within the global cropland planetary boundary appears to be realistic for Australians if they reduce their intake of discretionary foods to sensible levels and adjust food choices within the “meat and alternatives” food group. Importantly, from the perspective of cropland scarcity footprints, there appears to be no barrier to eating according to the current dietary guidelines. Our study has focused particularly on the 19–50-year age grouping which has the highest energy intake. Cropland scarcity footprints are lower for older age groups (Figure 1) and are expected to be lower again for many children. However, we have not considered food waste, which adds to cropland demand. It is also expected that continued progress in the agricultural sector in improving crop yields and livestock productivities will reduce the cropland intensity of food commodities and contribute to a downward trend in dietary cropland scarcity footprints over time.

### 4.2. Animal-Sourced Foods

Food systems can vary considerably in different regions of the world, making the comparison of results from different studies difficult. However, a consistent theme in the literature relating to dietary cropland requirements and dietary biodiversity impacts is the contribution from animal-sourced foods [25,39,42,46,55]. Our study of Australian daily diets showed that the “meat and alternatives” food group is important, but not as important as discretionary foods. Unfortunately, discretionary foods are rarely included in studies of sustainable diets [9], even recent prominent global assessments [4,47] including the EAT-Lancet Commission report [54]. However, discretionary foods can make up a large proportion of total energy intake (around one-third in Australia), so it is unsurprising that sustainable diet studies that overlook them overestimate the real contribution of other food groups, including lean meats. Discretionary foods can be of animal or plant origin, and they often combine ingredients from different sources. The important point is that they are not a necessary part of a healthy diet and should be eaten only occasionally and in small quantities [53]. They are suggested for variety and pleasure, not nutritional value. Therefore, reducing the intake of discretionary foods is not only beneficial to reducing cropland footprints, but also clearly beneficial to health.

Some discretionary foods have relatively high cropland scarcity footprints (Supplementary Table S3) and can exceed the cropland scarcity footprints of many core foods. Within the “meat and alternatives” food group, wild-caught seafood and game meats had no associated cropland use. However, the capacity to increase supplies of these food sources is limited in most cases. In the “meat and alternatives” food group, tofu had the next lowest cropland scarcity footprint (0.17 m^2^.yr-e per 170 g serving; Supplementary Table S3), along with pulses (0.18 m^2^.yr-e per 75 g serving). Beef and lamb had moderate cropland scarcity footprints (0.82 and 0.64 m^2^.yr-e per serving, respectively). In Australia, ruminant livestock are mostly raised on pasture and rangelands. For livestock production systems based predominantly on a feed ration, aquaculture salmon had the lowest cropland scarcity footprint (0.70 m^2^.yr-e per 100 g cooked fillet). The cropland scarcity footprints of eggs, chicken meat and pork meat were the highest (0.98, 1.62 and 2.21 m^2^.yr-e per serving, respectively). Dietary guidelines in Australia [53] emphasise the importance of enjoying a wide variety of nutritious foods as this is likely to ensure adequate intake of micronutrients. Our results show that Australians can choose a variety of foods within the “meat and alternatives” food group and eat within the global cropland planetary boundary, provided poultry and pork intake are moderated. This implies increased intake of other lean meats or foods of plant origin such as pulses or tofu.

### 4.3. Limitations

This study has examined cropland footprints of Australian adult daily diets. Other environmental aspects were not assessed, including the impacts related to the occupation of pastures and rangelands. As such, the results cannot be used to support conclusions about overall environmental sustainability. However, the same 9341 daily diets have previously been assessed for greenhouse gas emissions [61] and water scarcity footprint [49]. After controlling for energy intake, we found generally weak correlations between dietary greenhouse gas (GHG) emissions and dietary cropland footprints (Pearson correlation 0.20 to 0.36) as well as between dietary water scarcity footprints and dietary cropland footprints (Pearson correlation 0.21 to 0.25). Controlling for energy intake was undertaken because each of these environmental indicators has been found to be correlated with total dietary energy intake. What this means is that steps taken to reduce dietary cropland footprints may not always improve other environmental aspects and it may be a challenge to improve multiple environmental aspects concurrently. This needs to be explored further in future research.

Efforts were made to select the highest quality data sources. The dietary intake data from the Australian Health Survey is considered of high quality because of its large scale, representative sampling and systematic collection process. However, like all 24-h dietary recall surveys, there is uncertainty with regards to misreporting. Estimates of under-reporting were applied uniformly to the data. However, there is a possibility that under-reporting was biased towards discretionary foods. If so, the contribution of discretionary foods to cropland footprints may have been underestimated. An important innovation incorporated into this study was the application of impact factors from the field of life cycle assessment. Although there is uncertainty associated with the impact factors, we regard the results obtained after impact assessment to be more reliable in describing potential environmental burdens than results obtained without impact assessment. In this study, three cropland footprint indicators were used. Notwithstanding the uncertainties associated with any one indicator, the three sets of indicator results led to similar overall conclusions, giving confidence to study findings. Results obtained after impact assessment often differ greatly from results obtained prior to impact assessment [30,40], leading to different study conclusions. For example, several studies have noted that the biodiversity impacts of palm oil are disproportionately high relative to land area occupied [30,68,69]. Indeed, there is reason to question the fitness-for-purpose of sustainable diet studies that use “land area” as an indicator of environmental sustainability as “land area” does not reliably address relevant environmental concerns like cropland scarcity, biodiversity loss or malnutrition [27].

In summary, the average Australian adult diet currently has a cropland scarcity footprint that is too large for the proposed global cropland planetary boundary. However, diets in Australia vary greatly and a subgroup of healthier diets with cropland scarcity footprints well within the planetary boundary was identified, highlighting the important need to reduce the excessive consumption of discretionary foods. It is possible for Australians to meet current Australian Dietary Guidelines and remain within the cropland planetary boundary. However, this requires moderation of poultry and pork intake, with increased preference to seafood or other lean meats or foods of plant origin such as pulses or tofu.

## Figures and Tables

**Table 1:** Contribution of different foods (%) to the cropland scarcity footprint (CSF), cropland biodiversity footprint (CBF) and cropland malnutrition footprint (CMF) of Australian adult daily diets (*n* = 9341). Food groups are as defined by the Australian Dietary Guidelines.

Food	CSF	CBF	CMF
Fruit	9.5	9.9	9.3
Vegetables	5.0	5.3	4.5
Breads and cereals	12.6	11.2	11.9
Fresh meat and alternatives	27.4	23.9	26.5
Seafood	0.7	0.7	0.7
Beef and lamb	8.8	7.3	8.1
Poultry	11.7	9.5	11.4
Pork	2.9	2.5	2.8
Vegetarian alternatives	3.4	3.9	3.6
Other livestock products	<0.1	<0.1	<0.1
Dairy and alternatives	9.6	8.8	8.8
Beverages	1.5	3.8	3.8
Discretionary foods	32.7	35.6	33.6
Sugar-sweetened beverages	0.7	4.2	0.8
Biscuits, cakes, waffles	3.3	4.3	4.1
Pastries and pies	2.1	2.0	2.2
Processed meat products	12.5	10.1	11.9
Dairy desserts, cream, butter	2.0	2.2	2.0
Fried potato and extruded snacks	1.7	1.2	1.6
Muesli bars, confectionary, chocolate	2.5	4.7	4.4
Alcoholic beverages	6.0	5.5	5.0
Other	1.8	1.4	1.7
Healthy fats and oils	1.1	1.0	1.1
Miscellaneous foods	0.6	0.6	0.5

**Table 2:** Food intake (servings person^−1^) and cropland scarcity footprint (m^2^yr-e person^−1^) of Australian adult (19–50 years) daily diets.

Food Group	Higher Diet Quality/Lower Cropland Scarcity Footprint Subgroup (*n* = 824)		Lower Diet Quality/Higher Cropland Scarcity Footprint Subgroup (*n* = 832)	
	Servings	Cropland Scarcity Footprint	Servings	Cropland Scarcity Footprint
Fruit	1.53	0.60	0.91	0.46
Vegetables	3.37	0.37	1.47	0.24
Breads and cereals	5.45	0.76	3.95	0.82
Fresh meat and alternatives	1.92	1.20	2.62	3.18
Seafood	0.38	0.05	0.10	0.02
Beef and lamb	0.56	0.47	0.67	0.71
Poultry	0.24	0.41	1.18	1.79
Pork	0.03	0.04	0.28	0.43
Vegetarian alternatives	0.72	0.23	0.39	0.24
Dairy and alternatives	1.29	0.52	1.53	0.68
Discretionary foods	2.09	0.60	14.54	5.61
Miscellaneous foods		0.16		0.18
Total		4.21		11.17

**Table 3:** Food intake (servings person^−1^) and cropland scarcity footprint (m^2^yr-e person^−1^) for the current and recommended Australian adult (19–50 years) daily diets ^1^.

Food Group	Current Diet (*n* = 5157)		Recommended Diet Average Cropland Scarcity Footprint Intensity		Recommended Diet Improved Cropland Scarcity Footprint Intensity	
	Servings	Cropland Scarcity Footprint	Servings	Cropland Scarcity Footprint	Servings	Cropland Scarcity Footprint
Fruit	1.38	0.63	2.0	0.91	2.0	0.79
Vegetables	2.47	0.31	5.5	0.68	5.5	0.61
Breads and cereals	4.57	0.80	6.0	1.05	6.0	0.83
Fresh meat and alternatives	2.32	2.39	2.8	2.88	2.8	1.75
Seafood	0.22	0.04	0.27	0.04	0.56	0.07
Beef and lamb	0.66	0.60	0.79	0.73	0.81	0.69
Poultry	0.74	1.22	0.90	1.47	0.35	0.60
Pork	0.18	0.31	0.22	0.37	0.04	0.06
Vegetarian alternatives	0.51	0.22	0.61	0.27	1.04	0.33
Dairy and alternatives	1.46	0.63	2.5	1.07	2.5	1.00
Discretionary foods	7.42	2.48	2.8	0.94	2.8	0.81
Miscellaneous foods		0.17		0.17		0.17
Total		7.41		7.71		5.96

^1^ The improved cropland scarcity footprint intensity is based on the higher diet quality/lower cropland scarcity footprint subgroup. The number of servings differs marginally for men and women in the recommended Australian diet.

## Data Availability

The dietary intake data are available from the Australian Bureau of Statistics (http://www.abs.gov.au/australianhealthsurvey). Agricultural cropland footprint data are accessible at: https://article in press, citation to follow. The cropland footprints of individual foods are presented in the Supplementary Materials, Table S3.

## Supplementary Materials

The following are available online at https://www.mdpi.com/2072-6643/12/5/1212/s1, Table S1: Sample size by age and gender; Table S2: Number of 19–50-year-old adults in the quadrant analysis; Table S3: Cropland footprints of individual foods; Table S4: Cropland footprints of Australian adult diets by age group and gender; Table S5: Contribution of different foods (%) to the cropland scarcity footprint of Australian adult daily diets; Table S6: Contribution of different foods (%) to the cropland biodiversity footprint of Australian adult daily diets; Table S7: Contribution of different foods (%) to the cropland malnutrition footprint of Australian adult daily diets; Figure S1: Scatterplots showing diversity of individual adult daily diets; Table S8: Dietary intake and cropland biodiversity footprint; Table S9: Dietary intake and cropland malnutrition footprint; Table S10: Cropland biodiversity footprint of the current and recommended Australian adult (19 to 50 years) daily diet; Table S11: Cropland malnutrition footprint of the current and recommended Australian adult (19 to 50 years) daily diet.
